# Supplementary material for: Bacterial diet modulates tamoxifen-induced death via host fatty acid metabolism
Source: Nat Commun. 2022 Sep 23;13:5595. doi: 10.1038/s41467-022-33299-5 (PMC9508336; doi:10.1038/s41467-022-33299-5)
Supplement: Supplementary file 1 — Supplementary Information [file 41467_2022_33299_MOESM1_ESM.pdf]

Diot, et al. Supplementary Fig. 1

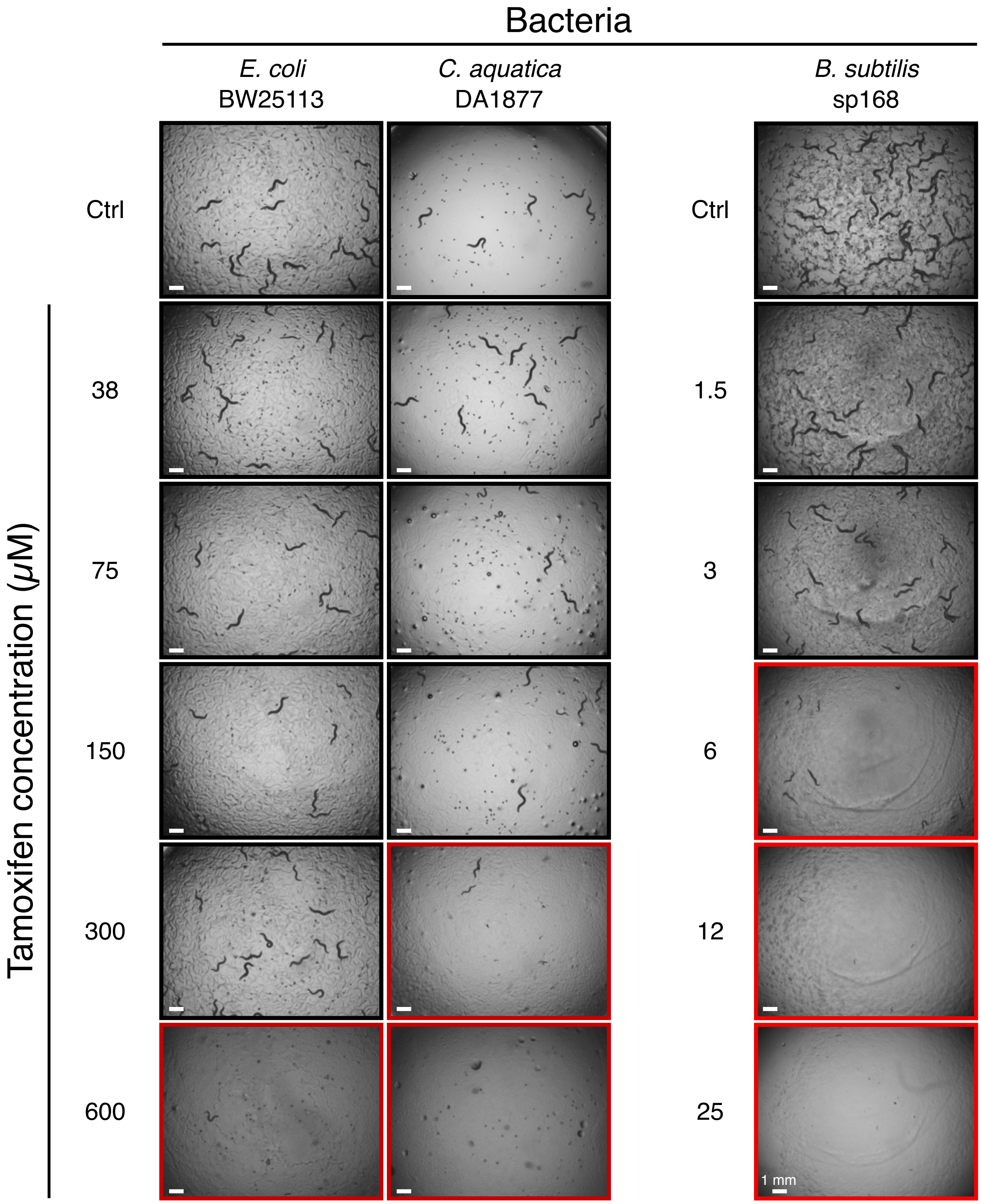

Supplementary Fig. 1: tamoxifen toxicity in non-arrested L1 animals

Bright-field images showing *C. elegans* supplemented with increasing doses of tamoxifen, fed *E. coli*, *C. aquatica*, and *B. subtilis*. In contrast to experiments presented in the main manuscript, experiments were conducted using *C. elegans* embryos rather than L1-arrested animals. Images were taken at 2x magnification after 48h exposure to tamoxifen. Scale bars: 1 mm. Representative results of 1 out of 3 independent experiments.

Diot, et al. Supplementary Fig. 2

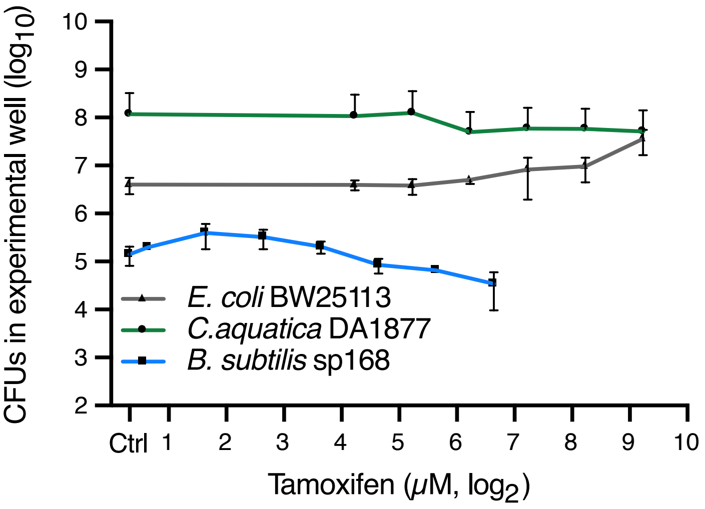

Supplementary Fig. 2: assessment of bacterial viability on tamoxifen containing NGM plates

Bacteria were seeded on tamoxifen containing NGM plates and incubated at room temperature for 48h. Bacterial lawns were recovered in M9 buffer, and colony-forming units were determined by plating serial dilutions on LB-agar plates. Data are represented as mean ±SEM of three independent biological replicates.

Source data are provided as a Source Data file.

Diot, *et al.* Supplementary Fig. 3

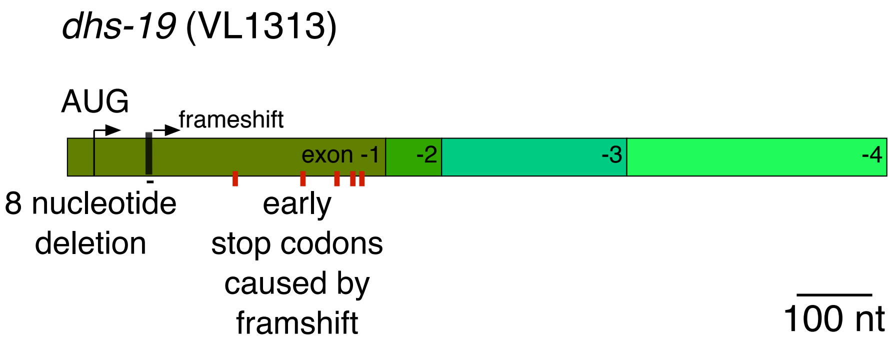

Supplementary Fig. 3: Schematic of the dhs-19 deletion

To-scale cartoon of the exon 1 frameshift caused by an 8-nucleotide deletion in the *dhs-19* gene, in the engineered VL1313 strain.

Diot, et al. Supplementary Fig. 4

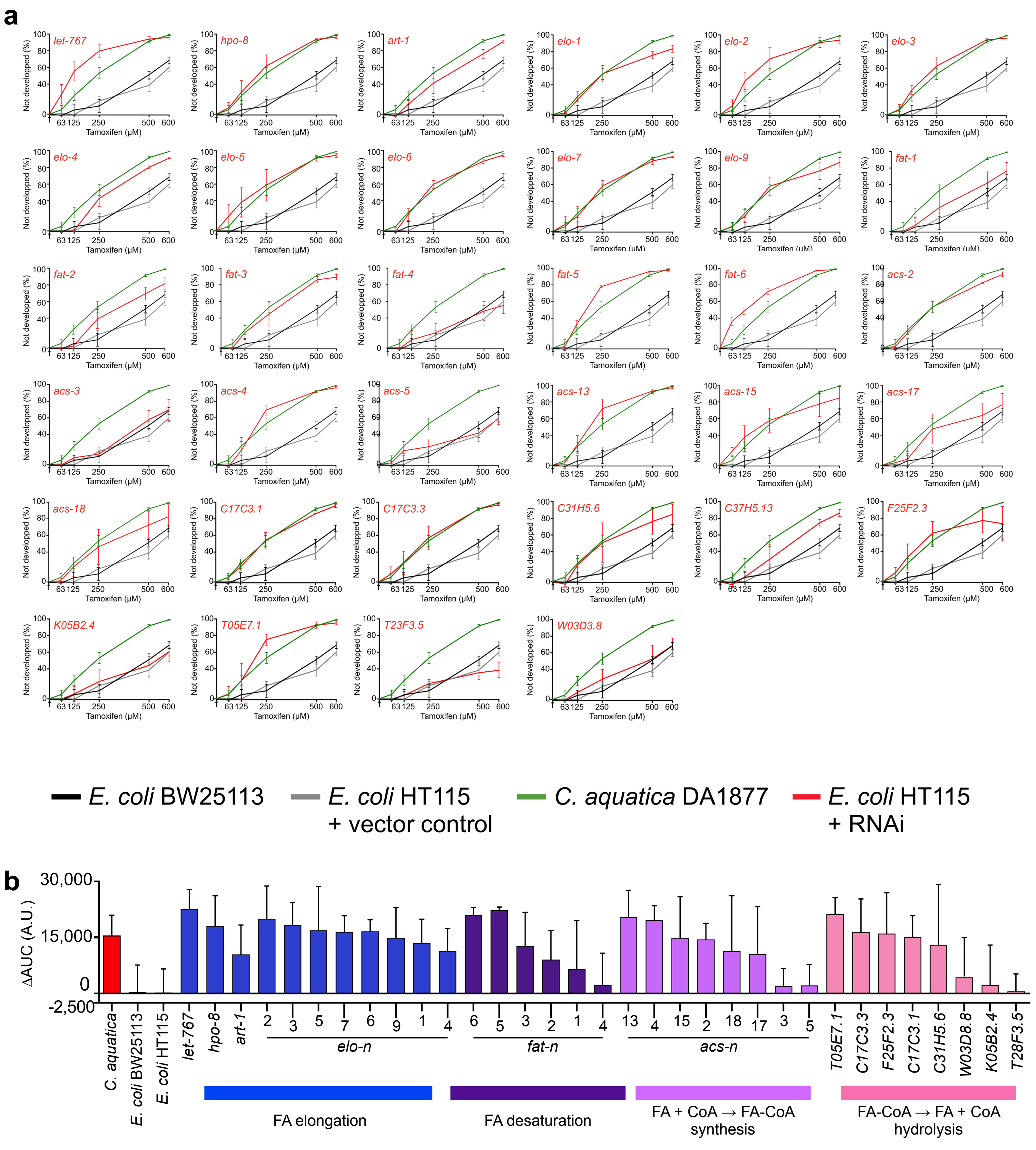

Supplementary Fig. 4: Individual dose-response curves for animals exposed to RNAi of genes involved in the fatty acid biosynthesis.

a All conditions were tested in parallel, and control conditions are plotted in each graph to facilitate the interpretation of the effect of individual RNAis. Data are represented as mean ±SEM of three biological replicates. Statistical significance was assessed by performing 2-way Anovas followed by a multi-comparison Dunett test using the no-RNAi *E. coli* condition as a control, on Graphpad Prism (v9). Adjusted *p-values* are reported in **Supplementary Table 1**.

b ΔAUCs presented in **Fig. 3e** with with standard errors. AUCs and error-bars were generated from the graphs in **a** using GraphPad Prism (v9). As AUC calculations derived a single value, the displayed ΔAUCs are representative of a single value. Standard error bars represent the standard error of the calculated AUC.

Source data are provided as a Source Data file.

Diot, et al. Supplementary Fig. 5

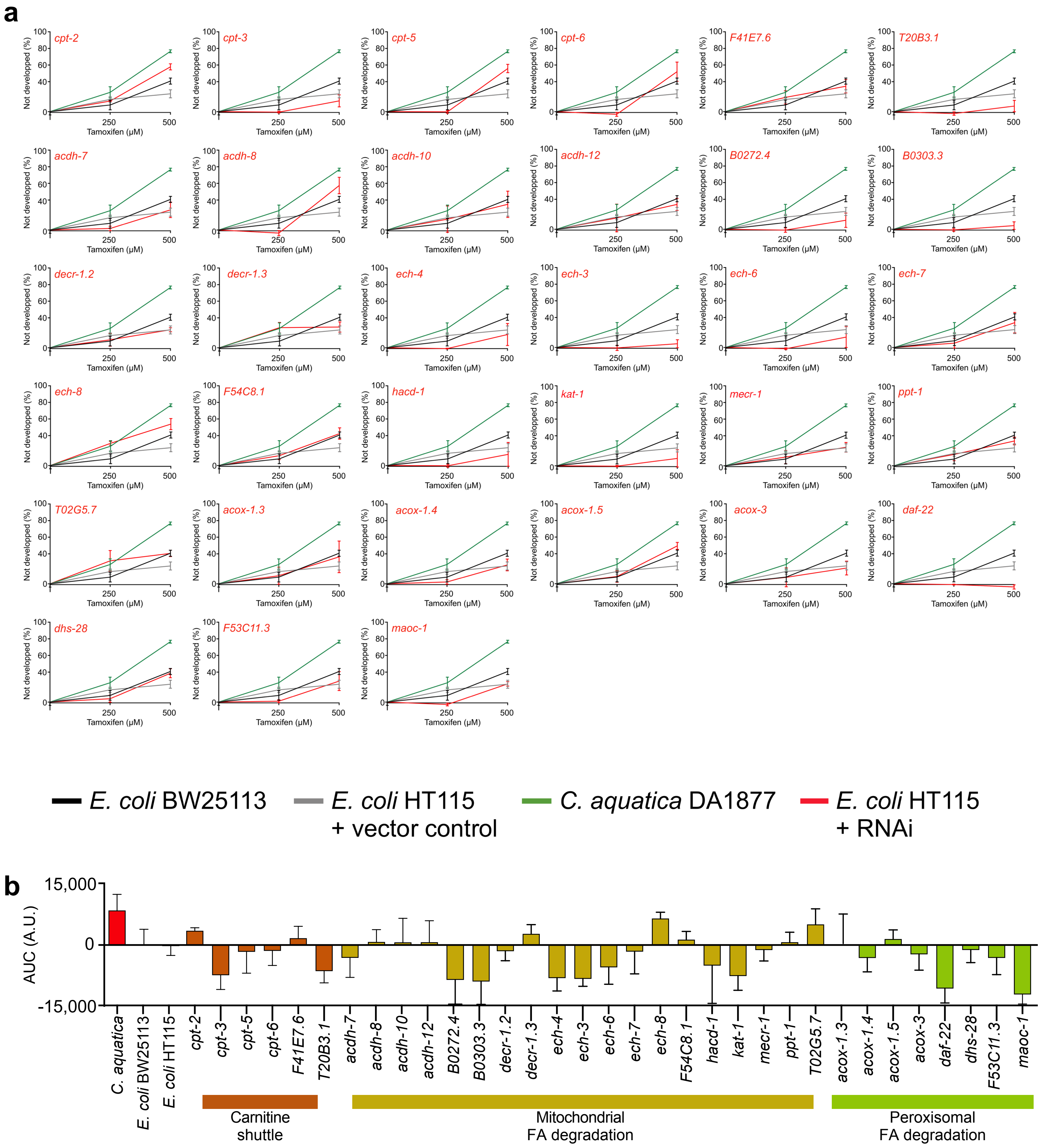

Supplementary Fig. 5: Individual dose-response curves for animals exposed to RNAi of genes involved in the fatty acid degradation.

a All conditions were tested in parallel, and control conditions are plotted in each graph to facilitate the interpretation of the effect of individual RNAis. Data are represented as mean ±SEM of three biological replicates. Statistical significance was assessed by performing 2-way Anovas followed by a multi-comparison Dunett test using the no-RNAi *E. coli* condition as a control, on Graphpad Prism (v9). Adjusted *p-values* are reported in **Supplementary Table 2**.

b ΔAUCs presented in **Fig. 3f** with standard errors. AUCs and error-bars were generated from the graphs in **a** using GraphPad Prism (v9). As AUC calculations derived a single value, the displayed ΔAUCs are representative of a single value. Standard error bars represent the standard error of the calculated AUC.

Source data are provided as a Source Data file.

Diot, et al. Supplementary Fig. 6

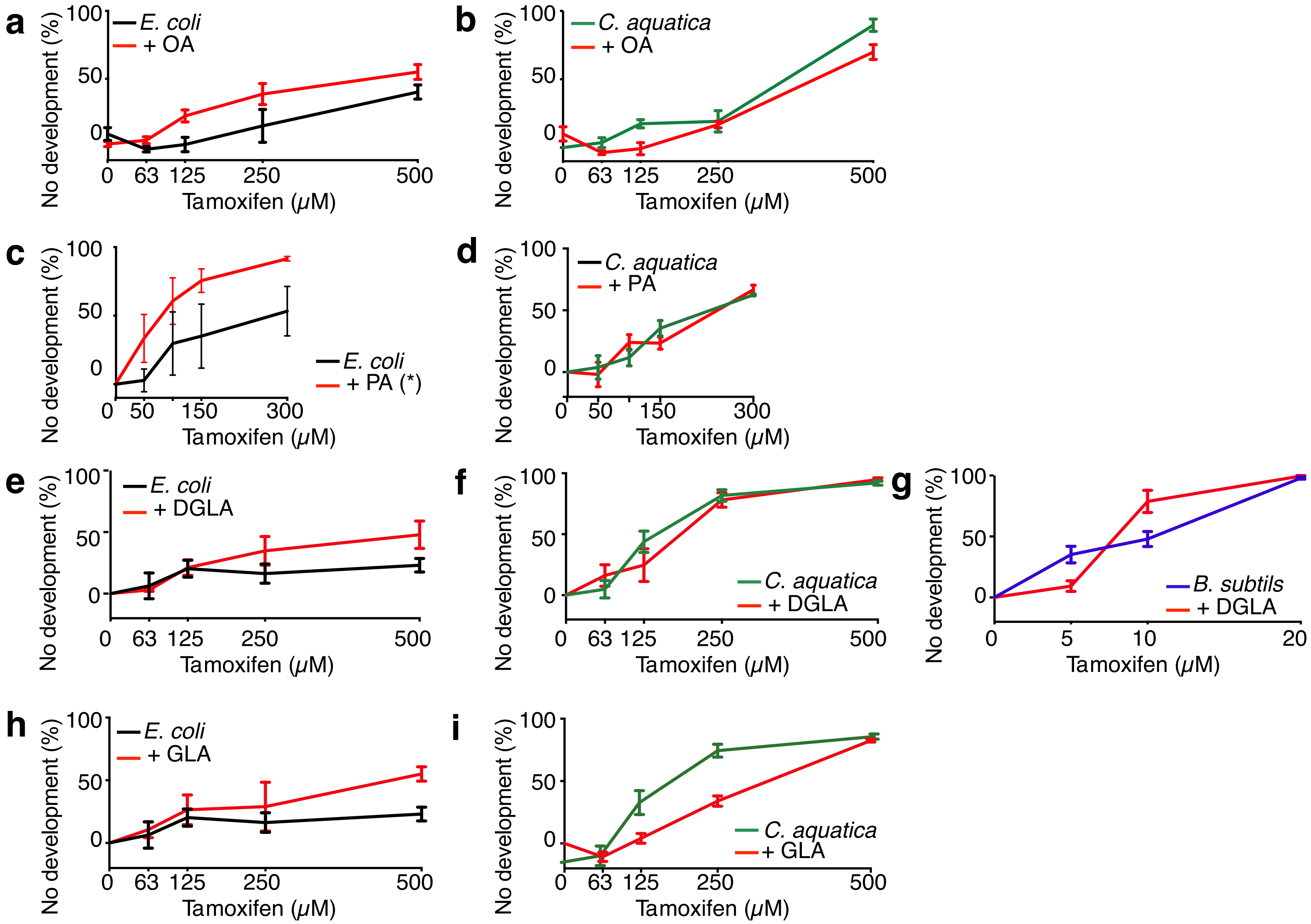

Supplementary Fig. 6: DRCs of tamoxifen toxicity with a larger panel of FA supplements.

a, **b** DRCs of tamoxifen toxicity with and without oleic acid (OA) supplementation on animals fed *E. coli* (**a**) or *C. aquatica* (**b**). *Adjusted p-values:* 0.6045 (**a**) and 0.0579 (**b**).

c, **d** DRCs of tamoxifen toxicity with and without palmitoleic acid (PA) supplementation on animals fed *E. coli* (**c**) or *C. aquatica* (**d**). *Adjusted p-values:* 0.0027 (**c**) and 0.9994 (**d**).

e-**g** DRCs of tamoxifen toxicity with and without di-homo-gamma-linoleic acid (DGLA) supplementation on animals fed *E. coli* (**e**), *C. aquatica* (**f**), or *B. subtilis* (**g**). *Adjusted p-values:* 0.3055 (**e**), 0.9974 (**f**), and 0.9857 (**g**).

h, **i** DRCs of tamoxifen toxicity with and without gamma-linoleic acid (GLA) supplementation on animals fed *E. coli* (**h**) or *C. aquatica* (**i**). *Adjusted p-values:* 0.0922 (**h**) and 0.0004 (**i**).

Data are represented as mean ±SEM of three independent biological replicates. Statistical significance was assessed by performing 2-way Anovas followed by a Dunett multi-comparison test using the non-supplemented conditions as controls, on Graphpad Prism (v9).

Source data are provided as a Source Data file.

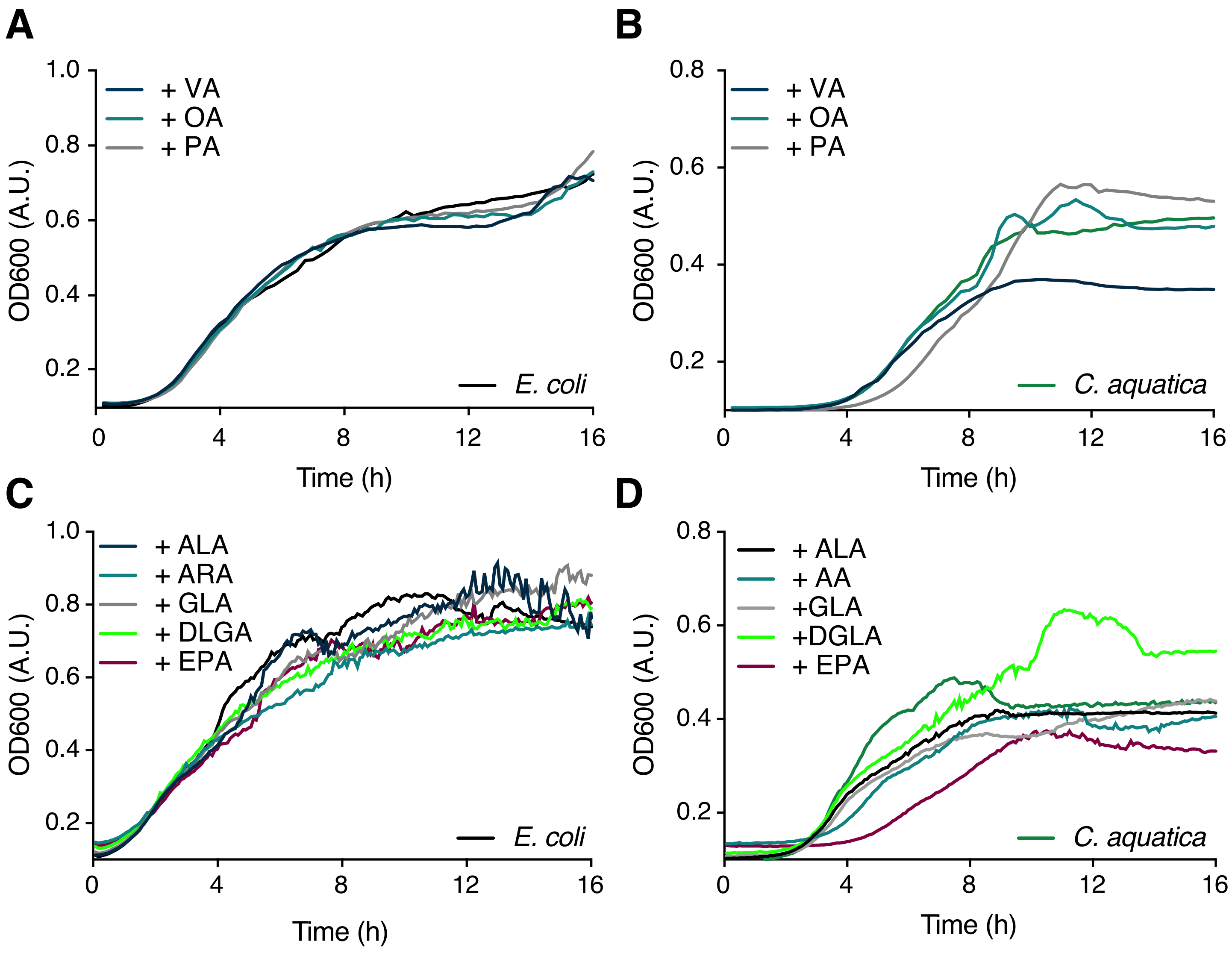
A
1.0
0.8
0.6
0.4
0.2
OD600 (A.U.)
+ VA
+ OA
+ PA
E. coli
0
4
8
12
16
Time (h)
B
0.8
0.6
0.4
0.2
OD600 (A.U.)
+ VA
+ OA
+ PA
C. aquatica
0
4
8
12
16
Time (h)
C
1.0
0.8
0.6
0.4
0.2
OD600 (A.U.)
+ ALA
+ ARA
+ GLA
+ DLGA
+ EPA
E. coli
0
4
8
12
16
Time (h)
D
0.8
0.6
0.4
0.2
OD600 (A.U.)
+ ALA
+ AA
+GLA
+DGLA
+ EPA
C. aquatica
0
4
8
12
16
Time (h)

Supplementary Fig. 7: Growth curves of *E. coli* and *C. aquatica* in presence of the FA supplements used in Fig. 4 and Supplementary Fig. S6.

a, **c** Growth curves of *E. coli* supplemented with MUFAs (A) and PUFAs (C). For clarity, no error bars are reported in the graphs. Data are represented as mean ±SEM of four independent biological replicates.

b, **d** Growth curves of *C. aquatica* supplemented with MUFAs (A) and PUFAs (C). For clarity, no error bars are reported in the graph. Data are represented as mean ±SEM of four independent biological replicates.

MUFA: mono-unsaturated FA, PUFA: poly-unsaturated FA, VA: cis-vaccenic acid, OA: oleic acid, PA: palmitoleic acid, ALA: alpha-linoleic acid, ARA: arachidonic acid, GLA: gamma-linoleic acid, DGLA: di-homo-gamma-linoleic acid, EPA: eicosapentaenoic acid.

Source data are provided as a Source Data file.

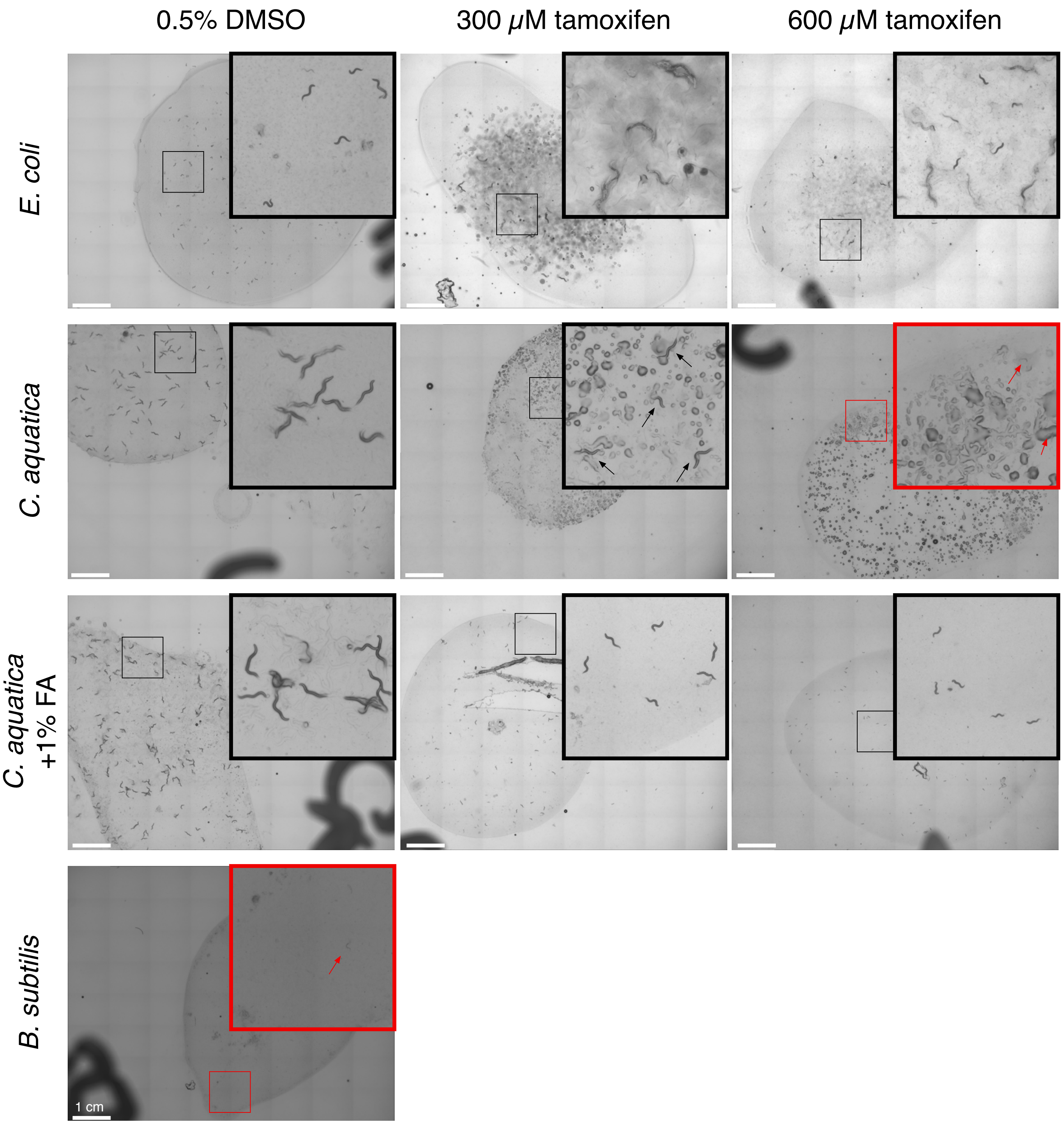
0.5% DMSO
300 μM tamoxifen
600 μM tamoxifen
E. coli
C. aquatica
C. aquatica +1% FA
B. subtilis
1 cm

Supplementary Fig. 8: tamoxifen toxicity in *C. elegans* fed metabolically inactive bacteria

Bacterial powders were seeded on tamoxifen containing NGM plates, supplemented with antibiotics and devoid of peptone, and with FA cocktail when indicated. Dried plates were then seeded with L1-synchronized and pictures were taken at 72h. Images were taken at 2x magnification after 48h exposure to tamoxifen. Scale bars: 1 cm. Representative results of 1 out of 3 independent experiments.

Diot, et al. Supplementary Fig. 9

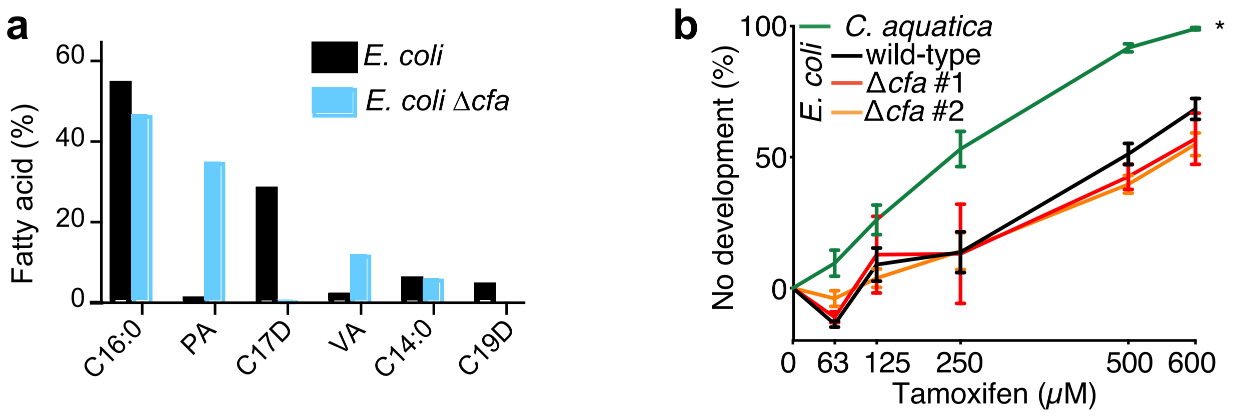

Supplementary Fig. 9: tamoxifen toxicity in C. elegans fed in ∆*cfa E. coli* mutant

a Fatty acid composition of *E. coli* BW25113 and ∆*cfa* mutant strains was assess by GC-MS. Statistical significance was assessed by performing unpaired two-tailed t-tests on Graphpad Prism (v9). *p*-values*:* 0.8717 (C16:0), <0.0001 (PA), 0.0007 (C17D), 0.0055 (VA), 0.0954 (C14:0), and 0.0027 (C17D).

b DRCs of tamoxifen toxicity on animals fed *C. aquatica*, *E. coli* BW25113, or *E. coli* ∆*cfa* mutant strains. Data are represented as mean ±SEM of three independent biological replicates. Statistical significance was assessed by performing 2-way Anovas followed by a Dunett multi-comparison test using the no-RNAi *E. coli* condition as control, on Graphpad Prism (v9). Adjusted *p*-values*:* <0.0001 (*C. aquatica*), >0.9999 (∆*cfa*#1), >0.9999 (∆*cfa*#2).

Source data are provided as a Source Data file.

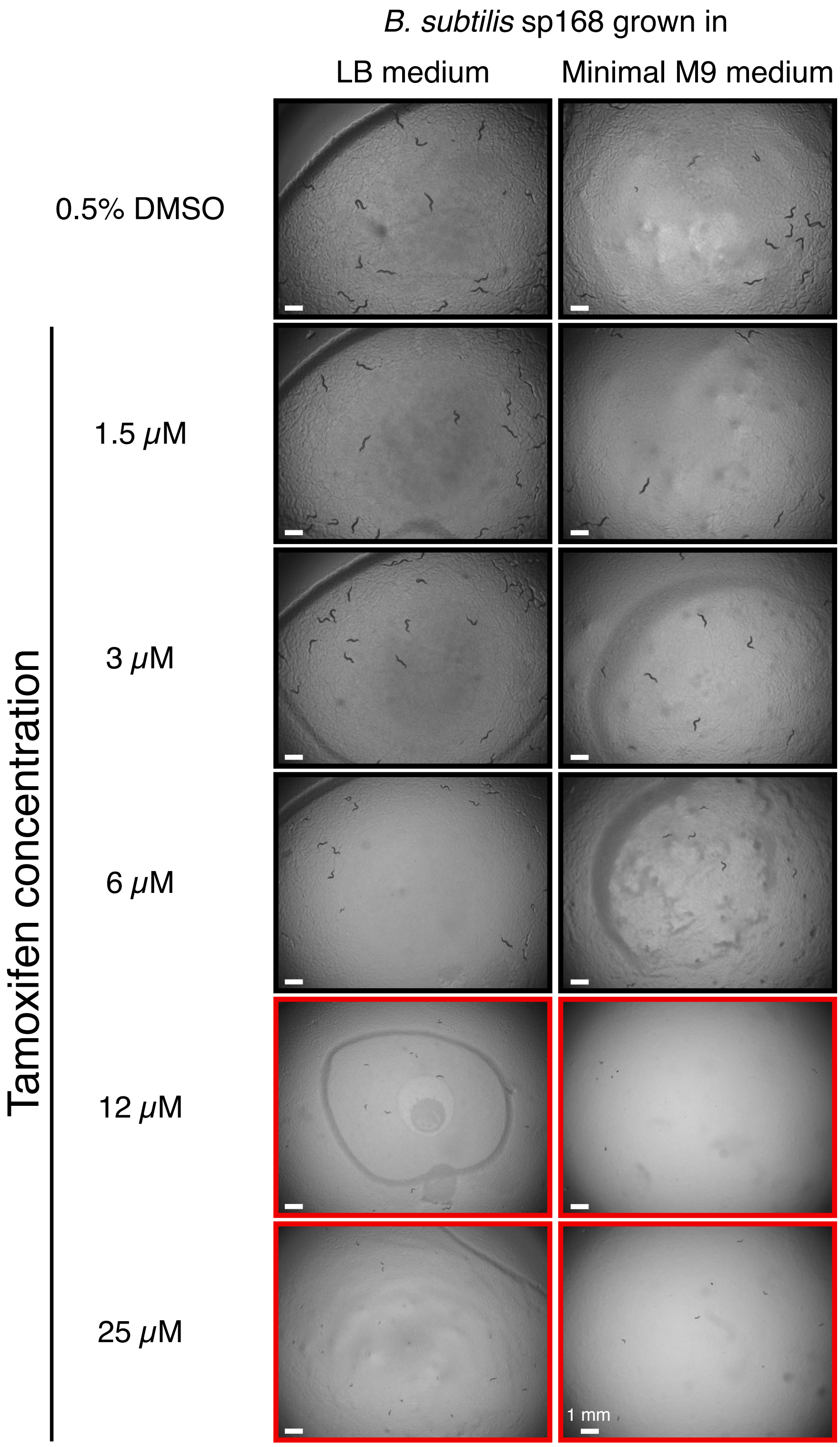
B. subtilis sp168 grown in
LB medium
Minimal M9 medium
0.5% DMSO
Tamoxifen concentration
1.5 μM
3 μM
6 μM
12 μM
25 μM
1 mm

Supplementary Fig. 10: tamoxifen toxicity in *C. elegans* fed *B. subtilis* grown in M9 minimal or LB media

Bright-field images showing *C. elegans* supplemented with increasing doses of tamoxifen, fed *B. subtilis* grown in LB or M9 minimal media supplemented with glucose. Scale bars: 1 mm. Images were taken at 2x magnification after 48h exposure to tamoxifen.

Representative results of 1 out of 3 independent experiments.

Diot, et al. Supplementary Fig. 11

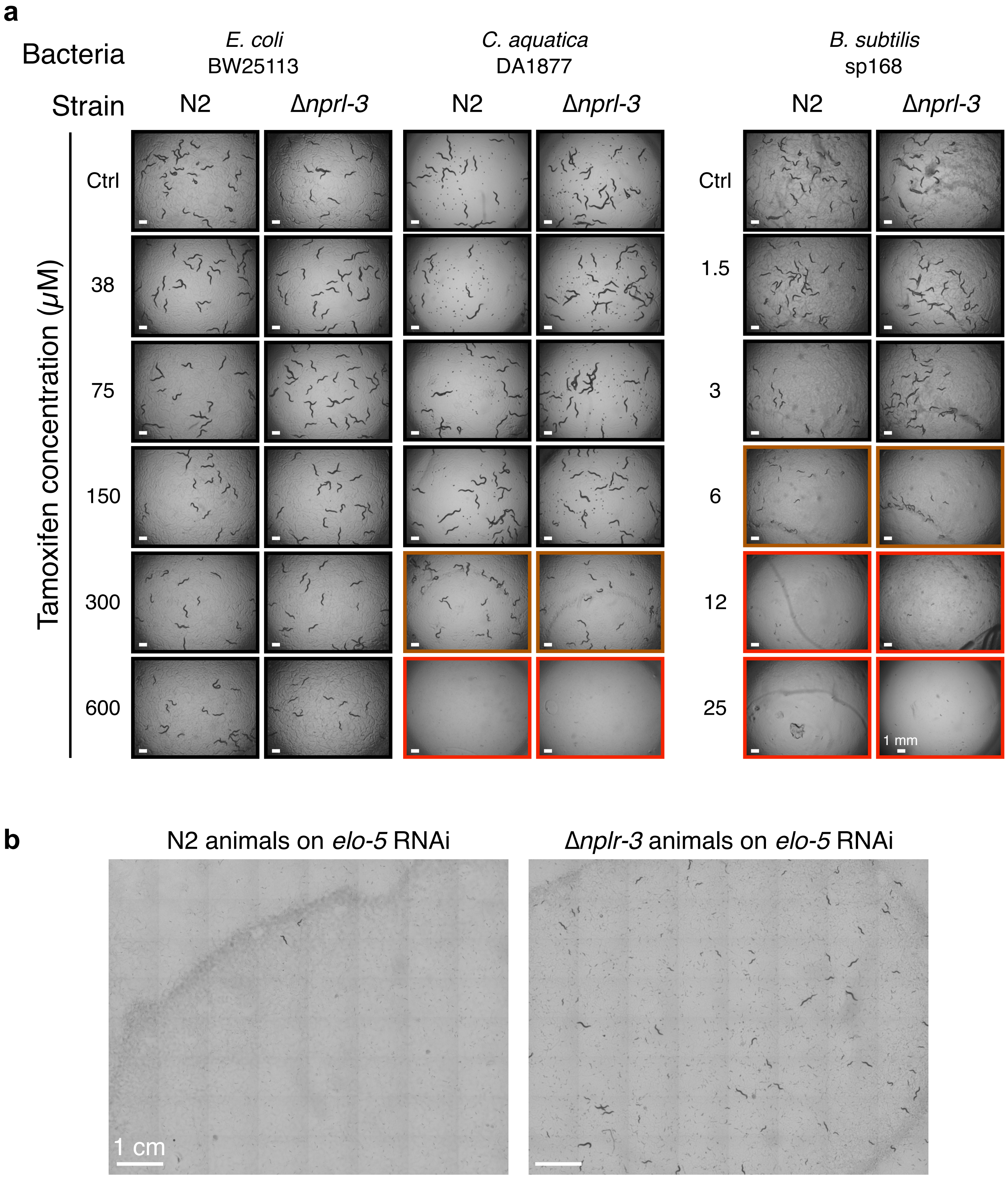

Supplementary Fig. 11: tamoxifen toxicity in ∆*nprl-3* animals

a-b experiments were conducted in parallel. Images are representative of three independent experiments.

a Bright-field images showing N2 and ∆*nprl-3* animal strains supplemented with increasing doses of tamoxifen, fed *E. coli, C. aquatica or B. subtilis.* Images were taken at 2x magnification after 48h exposure to tamoxifen. Scale bars: 1 mm.

b Bright-field images showing N2 and ∆*nprl-3* animal strains fed *E. coli* expressing double stranded RNA as indicated. F0 showed no phenotype. At 72h adult animals were removed from the plate and progeny was monitored daily. Images were taken at 10x magnification after 120h (48h post F0 removal). Scale bars: 1 cm.

Representative results of 1 out of 3 independent experiments.

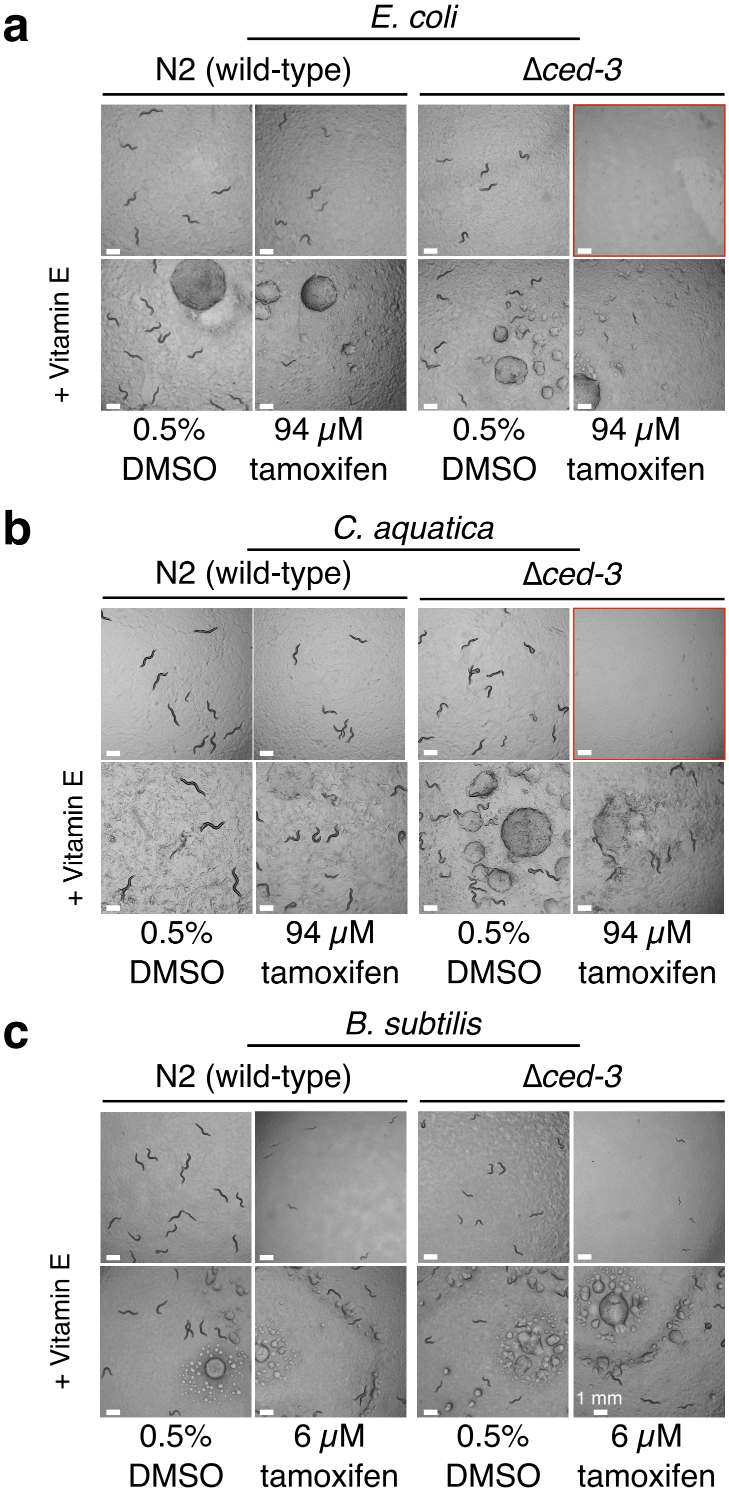
a
E. coli
N2 (wild-type)
Δced-3
+ Vitamin E
0.5% DMSO
94 μM tamoxifen
0.5% DMSO
94 μM tamoxifen
b
C. aquatica
N2 (wild-type)
Δced-3
+ Vitamin E
0.5% DMSO
94 μM tamoxifen
0.5% DMSO
94 μM tamoxifen
c
B. subtilis
N2 (wild-type)
Δced-3
+ Vitamin E
1 mm
0.5% DMSO
6 μM tamoxifen
0.5% DMSO
6 μM tamoxifen

Supplementary Fig. 12: diet-dependent potentiation of tamoxifen toxicity by a genetic deficiency to engage apoptosis

a Bright-field images (left) of wild type and ∆ced-3 animals fed E. coli supplemented or not with Vitamin E.

b Bright-field images (left) of wild type and ∆ced-3 animals fed C. aquatica supplemented or not with Vitamin E.

c Bright-field images (left) of wild type and ∆ced-3 animals fed B. subtilis supplemented or not with Vitamin E.

Scale bars: 1 mm. Representative results of 1 out of 3 independent experiments.

Diot, et al. Supplementary Fig. 13

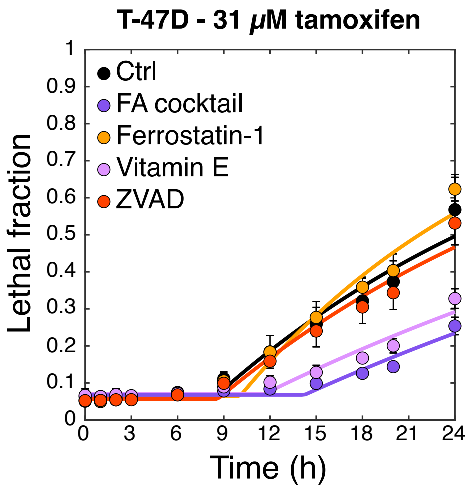

Supplementary Fig. 13: effect of supplements on tamoxifen toxicity in ER-positive T-47D breast cancer cells

Fractional viability of ER-positive T-47D breast cancer cells plotted as a function of time, in presence of the indicated supplements. Data are represented as mean ±SD of four technical replicates. Representative results of 1 out 3 independent experiments.

Source data are provided as a Source Data file.

Supplementary Table 1

Results of statistical analysis of tamoxifen dose response curves on animals knocked-down for genes involved in fatty acid bionsynthesis

Control	Condition	p-value	Significance	Figure
E. coli	*C. aquatica*	<0.0001	****	Supplemental Figure 1
E. coli	*let-727*	<0.0001	****	Supplemental Figure 1
E. coli	*hpo-8*	<0.0001	****	Supplemental Figure 1
E. coli	*art-1*	0.0021	ns	Supplemental Figure 1
E. coli	*elo-1*	0.0001	***	Supplemental Figure 1
E. coli	*elo-2*	<0.0001	****	Supplemental Figure 1
E. coli	*elo-3*	<0.0001	****	Supplemental Figure 1
E. coli	*elo-4*	0.0171	ns	Supplemental Figure 1
E. coli	*elo-5*	<0.0001	****	Supplemental Figure 1
E. coli	*elo-6*	<0.0001	****	Supplemental Figure 1
E. coli	*elo-7*	<0.0001	****	Supplemental Figure 1
E. coli	*elo-9*	<0.0001	****	Supplemental Figure 1
E. coli	*fat-1*	>0.9999	ns	Supplemental Figure 1
E. coli	*fat-2*	0.311	ns	Supplemental Figure 1
E. coli	*fat-3*	<0.0001	****	Supplemental Figure 1
E. coli	*fat-4*	>0.9999	ns	Supplemental Figure 1
E. coli	*fat-5*	<0.0001	****	Supplemental Figure 1
E. coli	*fat-6*	<0.0001	****	Supplemental Figure 1
E. coli	*acs-2*	<0.0001	****	Supplemental Figure 1
E. coli	*acs-3*	>0.9999	ns	Supplemental Figure 1
E. coli	*acs-4*	<0.0001	****	Supplemental Figure 1
E. coli	*acs-5*	>0.9999	ns	Supplemental Figure 1
E. coli	*acs-13*	<0.0001	****	Supplemental Figure 1
E. coli	*acs-15*	<0.0001	****	Supplemental Figure 1
E. coli	*acs-17*	0.0522	ns	Supplemental Figure 1
E. coli	*acs-18*	0.001	ns	Supplemental Figure 1
E. coli	*C17C3.1*	<0.0001	****	Supplemental Figure 1
E. coli	*C17C3.3*	<0.0001	****	Supplemental Figure 1
E. coli	*C31H5.6*	0.0002	***	Supplemental Figure 1
E. coli	*F25F2.3*	<0.0001	****	Supplemental Figure 1
E. coli	*K05B2.4*	>0.9999	ns	Supplemental Figure 1
E. coli	*T05E7.1*	<0.0001	****	Supplemental Figure 1
E. coli	*T28F3.5*	>0.9999	ns	Supplemental Figure 1
E. coli	*W03D8.8*	>0.9999	ns	Supplemental Figure 1

Supplementary Table 2

Results of statistical analysis of tamoxifen dose response curves on animals knocked-down for genes involved in fatty acid degradation

Control	Condition	p-value	Significance	Figure
E. coli	*C. aquatica*	0.0004	***	Supplemental Figure 2
E. coli	*cpt-2*	0.9985	ns	Supplemental Figure 2
E. coli	*cpt-3*	0.5142	ns	Supplemental Figure 2
E. coli	*cpt-5*	>0.9999	ns	Supplemental Figure 2
E. coli	*cpt-6*	>0.9999	ns	Supplemental Figure 2
E. coli	*F41E7.6*	0.9999	ns	Supplemental Figure 2
E. coli	*T20B3.1*	0.5586	ns	Supplemental Figure 2
E. coli	*acdh-7*	0.9988	ns	Supplemental Figure 2
E. coli	*acdh-8*	0.9997	ns	Supplemental Figure 2
E. coli	*acdh-10*	>0.9999	ns	Supplemental Figure 2
E. coli	*B0272.4*	0.2354	ns	Supplemental Figure 2
E. coli	*B0303.3*	0.1241	ns	Supplemental Figure 2
E. coli	*decr-1.2*	0.9992	ns	Supplemental Figure 2
E. coli	*decr-1.3*	0.9998	ns	Supplemental Figure 2
E. coli	*ech-4*	0.4453	ns	Supplemental Figure 2
E. coli	*ech-3*	0.1871	ns	Supplemental Figure 2
E. coli	*ech-6*	0.8801	ns	Supplemental Figure 2
E. coli	*ech-7*	0.9994	ns	Supplemental Figure 2
E. coli	*ech-8*	0.9489	ns	Supplemental Figure 2
E. coli	*F54C8.1*	0.9997	ns	Supplemental Figure 2
E. coli	*hacd-1*	0.9423	ns	Supplemental Figure 2
E. coli	*kat-1*	0.3731	ns	Supplemental Figure 2
E. coli	*mecr-1*	0.9993	ns	Supplemental Figure 2
E. coli	*ppt-1*	>0.9999	ns	Supplemental Figure 2
E. coli	*T02G5.7*	0.9987	ns	Supplemental Figure 2
E. coli	*acox-1.3*	0.9999	ns	Supplemental Figure 2
E. coli	*acox-1.4*	0.9986	ns	Supplemental Figure 2
E. coli	*acox-1.5*	0.9995	ns	Supplemental Figure 2
E. coli	*acox-3*	0.9988	ns	Supplemental Figure 2
E. coli	*daf-22*	0.0141	*	Supplemental Figure 2
E. coli	*dhs-28*	0.9997	ns	Supplemental Figure 2
E. coli	*F53C11.3*	0.9988	ns	Supplemental Figure 2
E. coli	*maoc-1*	0.0594	ns	Supplemental Figure 2
E. coli	*W03D8.8*	>0.9999	ns	Supplemental Figure 1

Supplementary Table 3

Bacterial fatty acid composition. Results are expressed as the mean ± SD of three biological replicates.

			E. coli	*E. coli* + 175 μM TAM	*C. aquatica*	*C. aquatica* + 175 μM TAM	*B. subtilis*
CPFA		C17D	31.84 ± 5.56	31.65 ± 1.86	3.78 ± 1.18	5.59 ± 2.26	not detected
CPFA		C19D	13.85 ± 3.47	13.79 ± 0.92	0.84 ± 0.82	0.38 ± 0.37	not detected
mmBCFA		C15iso	not detected	0.19 ± 0.19	0.01 ± 0.02	0.02 ± 0.02	15.52 ± 3.04
mmBCFA		C15ante-iso	not detected	not detected	not detected	not detected	70.76 ± 2.66
mmBCFA		C17iso	0.02 ± 0.04	0.02 ± 0.03	0.16 ± 0.28	0.06 ± 0.1	2.49 ± 0.46
mmBCFA		C17ante-iso	not detected	not detected	not detected	not detected	7.35 ± 1.08
MUFA	Palmitoleic Acid	C16:1n7	4.22 ± 3.77	4.41 ± 3.94	41.65 ± 9.06	44.54 ± 3.31	1.28 ± 0.13
MUFA	Oleic Acid	C18:1n9	5.74 ± 3.18	5.58 ± 3.14	5.37 ± 1.16	8.62 ± 2.19	not detected
MUFA	Vaccenic Acid	C18:1n7	0.9 ± 1.55	0.71 ± 1.24	not detected	0.13 ± 0.22	not detected
SFA	Myristic Acid	C14:0	8.27 ± 0.57	8.05 ± 0.86	3.95 ± 0.83	4.00 ± 0.01	0.3 ± 0.01
SFA	Palmitic Acid	C16:0	34.2 ± 6.74	34.93 ± 3.44	37.07 ± 6.29	36.29 ± 3.63	1.45 ± 0.27
SFA	Stearic Acid	C18:0	0.55 ± 0.15	0.48 ± 0.14	0.68 ± 0.69	0.37 ± 0.36	not detected
	Total CPFA		45.69 ± 8.66	45.44 ± 0.96	4.62 ± 1.97	5.97 ± 2.08	not detected
	Total mmBCFA		0.02 ± 0.04	0.2 ± 0.22	0.18 ± 0.31	0.08 ± 0.12	96.57 ± 0.19
	Total MUFA		10.86 ± 3.53	10.71 ± 4.17	47.02 ± 9.88	53.29 ± 2.48	1.28 ± 0.13
	Total PUFA		not detected	not detected	not detected	not detected	not detected
	Total SFA		43.02 ± 7.41	43.47 ± 3.25	41.69 ± 7.18	40.66 ± 3.51	2.11 ± 0.33

Supplementary Table 4

Fatty acid composition of *C. elegans*. Results are expressed as the mean ± SD of three biological replicates.

Condition			*E. coli* BW25113	*E. coli* BW25113 + 175 µM tamoxifen	*E. coli* HT115 + Control RNAi	*E. coli* HT115 + 175 µM tamoxifen + Control RNAi	*C. aquatica*	*C. aquatica* + 175 µM tamoxifen	*C. aquatica* + FA cocktail	*C. aquatica* + 175 µM tamoxifen + FA cocktail	*B. subtilis*
CPFA	C17D		07.43% ± 0.73	07.63% ± 0.43	04.73% ± 1.81	04.68% ± 2.03	00.89% ± 0.23	01.02% ± 0.33	00.53% ± 0.12	00.90% ± 0.24	0.79 ± 0.01
CPFA	C19D		04.59% ± 0.18	04.85% ± 0.40	01.79% ± 0.92	01.92% ± 0.87	00.31% ± 0.06	00.17% ± 0.15	00.08% ± 0.08	00.12% ± 0.10	not detected
mmBCFA	C15iso		02.13% ± 0.53	02.03% ± 0.49	01.41% ± 0.26	01.31% ± 0.28	01.56% ± 0.15	01.60% ± 0.25	01.07% ± 0.11	01.18% ± 0.08	2.01 ± 0.08
mmBCFA	C15-anteiso		not detected	not detected	not detected	not detected	not detected	not detected	not detected	not detected	8.38 ± 0.24
mmBCFA	C17iso		05.86% ± 0.46	06.44% ± 0.44	04.28% ± 1.42	04.68% ± 1.72	04.19% ± 0.40	04.42% ± 0.20	02.95% ± 0.23	02.87% ± 0.43	7.43 ± 0.07
mmBCFA	C17-anteiso		not detected	not detected	not detected	not detected	not detected	not detected	not detected	not detected	16.93 ± 0.27
MUFA	Palmitoleic Acid	C16:1n7	00.46% ± 0.05	00.38% ± 0.04	01.08% ± 0.21	00.84% ± 0.12	05.65% ± 0.96	05.06% ± 1.53	03.35% ± 1.37	03.48% ± 0.23	0.61 ± 0.01
MUFA	Oleic Acid	C18:1n9	04.48% ± 1.42	03.74% ± 0.05	03.61% ± 1.40	03.55% ± 1.55	04.06% ± 0.06	04.04% ± 0.11	01.26% ± 0.44	01.36% ± 0.36	2.59 ± 0.26
MUFA	Vaccenic Acid	C18:1n7	18.37% ± 1.32	17.16% ± 2.16	24.96% ± 8.64	24.78% ± 9.57	33.33% ± 1.60	34.16% ± 3.83	43.82% ± 0.94	45.14% ± 1.63	0.54 ± 0.07
PUFA	γ-Linolenic acid	C18:3γ	01.08% ± 0.94	01.13% ± 0.99	00.71% ± 0.30	00.60% ± 0.28	00.70% ± 0.61	00.74% ± 0.41	00.29% ± 0.08	00.42% ± 0.09	2.51 ± 0.19
PUFA	Linoleic Acid	C18:2n6	10.25% ± 0.34	11.02% ± 0.23	09.82% ± 0.90	10.04% ± 1.69	09.90% ± 0.17	09.64% ± 0.14	04.22% ± 1.13	04.74% ± 1.35	4.65 ± 0.39
PUFA	Arachadinoc Acid	C20:4n6	01.19% ± 0.02	01.33% ± 0.12	01.08% ± 0.38	01.15% ± 0.37	01.18% ± 0.27	01.15% ± 0.27	00.44% ± 0.09	00.55% ± 0.09	2.47 ± 0.06
PUFA	Eicosapentaenoic acid	C20:5n3	23.32% ± 1.07	23.83% ± 0.88	28.87% ± 9.09	28.98% ± 8.33	21.41% ± 0.48	20.92% ± 1.38	23.97% ± 3.15	24.27% ± 1.14	29.26 ± 0.58
PUFA	Dihomo-γ-linolenic acid	C20:3n6	03.84% ± 0.19	03.88% ± 0.31	02.75% ± 0.73	02.68% ± 0.81	02.67% ± 0.24	02.51% ± 0.39	02.00% ± 0.12	02.15% ± 0.16	6.53 ± 0.02
PUFA	Eicosatetraenoic acid	C20:4n3	05.54% ± 0.12	05.38% ± 0.37	05.03% ± 1.07	04.94% ± 1.30	03.76% ± 0.20	03.55% ± 0.59	04.99% ± 0.47	04.47% ± 0.15	7.48 ± 0.24
SFA	Myristic Acid	C14:0	0.27% ± 0.04	0.24% ± 0.04	00.19% ± 0.06	00.19% ± 0.08	00.11% ± 0.01	00.13% ± 0.02	00.09% ± 0.04	00.1% ± 0.01	0.31 ± 0.09
SFA	Palmitic Acid	C16:0	03.68% ± 0.42	03.00% ± 0.62	03.27% ± 1.15	02.82% ± 0.67	03.17% ± 0.22	03.10% ± 0.51	03.05% ± 1.16	02.45% ± 0.20	1.48 ± 0.38
SFA	Stearic Acid	C18:0	06.81% ± 0.80	07.18% ± 0.54	05.80% ± 1.47	06.50% ± 1.92	06.88% ± 1.24	07.56% ± 0.67	07.73% ± 1.08	05.59% ± 1.04	3.17 ± 0.37
**Total CPFA**			12.02 ± 0.85	12.48 ± 0.83	6.51 ± 2.72	6.6 ± 2.83	1.2 ± 0.17	1.19 ± 0.37	0.61 ± 0.2	1.02 ± 0.34	0.79 ± 0.01
**Total mmBCFA**			7.99 ± 0.97	8.47 ± 0.93	5.68 ± 1.5	5.98 ± 1.67	5.75 ± 0.51	6.03 ± 0.07	4.01 ± 0.31	4.05 ± 0.43	34.76 ± 0.31
**Total MUFA**			23.31 ± 0.18	21.28 ± 2.14	29.65 ± 8.77	29.16 ± 9.4	43.04 ± 2.05	43.27 ± 2.35	48.43 ± 0.94	49.98 ± 1.04	3.74 ± 0.32
**Total PUFA**			45.92 ± 1.12	47.35 ± 0.97	48.89 ± 8.44	48.74 ± 8.75	39.86 ± 1.09	38.72 ± 3.05	36.07 ± 2.46	36.81 ± 2.07	52.89 ± 0.85
**Total SFA**			10.75 ± 1.03	10.42 ± 0.39	9.26 ± 2.47	9.51 ± 2.44	10.16 ± 1.04	10.79 ± 0.87	10.87 ± 2.06	8.14 ± 0.93	7.82 ± 0.91
