# Supplementary material for: Bacterial diet modulates tamoxifen-induced death via host fatty acid metabolism
Source: Nat Commun. 2022 Sep 23;13:5595. doi: 10.1038/s41467-022-33299-5 (PMC9508336; doi:10.1038/s41467-022-33299-5)
Supplement: Supplementary file 2 — Description of Additional Supplementary Files [file 41467_2022_33299_MOESM2_ESM.pdf]

Description of Additional Supplementary Files

File Name: Supplementary Data 1

Description: Pair-wise statistical comparisons of the fatty acid species measured in *C. elegans* fed *E. coli* and *C. aquatica*. Statistical significance was assessed by performing two-tailed *t*-tests on GraphPad Prism (v9).

File Name: Supplementary Data 2

Description: List of differentially expressed genes in *C. elegans* fed *E. coli* and exposed to a 400 µM tamoxifen dose (p-value<0.01; fold-change>2). Up- and down-regulated genes are separated in two distinct sub-tables. Differentially expressed genes were identified using DEseq2, p-values were obtained using the Wald test, and adjusted following the Benjamini-Hochberg method.
