# Supplementary material for: Bacterial diet modulates tamoxifen-induced death via host fatty acid metabolism
Source: Nat Commun. 2022 Sep 23;13:5595. doi: 10.1038/s41467-022-33299-5 (PMC9508336; doi:10.1038/s41467-022-33299-5)
Supplement: Supplementary file 5 — Reporting Summary [file 41467_2022_33299_MOESM5_ESM.pdf]

nature portfolio

Corresponding author(s): Albertha J. M. Walhout

Last updated by author(s): Aug 22, 2022

Reporting Summary

Nature Portfolio wishes to improve the reproducibility of the work that we publish. This form provides structure for consistency and transparency in reporting. For further information on Nature Portfolio policies, see our Editorial Policies and the Editorial Policy Checklist.

Statistics

For all statistical analyses, confirm that the following items are present in the figure legend, table legend, main text, or Methods section.

n/a	Confirmed	
☐	☒	The exact sample size (*n*) for each experimental group/condition, given as a discrete number and unit of measurement
☐	☒	A statement on whether measurements were taken from distinct samples or whether the same sample was measured repeatedly
☐	☒	The statistical test(s) used AND whether they are one- or two-sided<br>*Only common tests should be described solely by name; describe more complex techniques in the Methods section.*
☒	☐	A description of all covariates tested
☒	☐	A description of any assumptions or corrections, such as tests of normality and adjustment for multiple comparisons
☐	☒	A full description of the statistical parameters including central tendency (e.g. means) or other basic estimates (e.g. regression coefficient) AND variation (e.g. standard deviation) or associated estimates of uncertainty (e.g. confidence intervals)
☐	☒	For null hypothesis testing, the test statistic (e.g. *F*, *t*, *r*) with confidence intervals, effect sizes, degrees of freedom and *P* value noted<br>*Give P values as exact values whenever suitable.*
☒	☐	For Bayesian analysis, information on the choice of priors and Markov chain Monte Carlo settings
☒	☐	For hierarchical and complex designs, identification of the appropriate level for tests and full reporting of outcomes
☒	☐	Estimates of effect sizes (e.g. Cohen's *d*, Pearson's *r*), indicating how they were calculated

Our web collection on statistics for biologists contains articles on many of the points above.

Software and code

Policy information about availability of computer code

Data collection	No software was used for data collection.
Data analysis	Graphs and statistics were realized on GraphPad Prism (v9).<br>RNAseq data were analyzed using a homemade DolphinNext pipeline.

For manuscripts utilizing custom algorithms or software that are central to the research but not yet described in published literature, software must be made available to editors and reviewers. We strongly encourage code deposition in a community repository (e.g. GitHub). See the Nature Portfolio guidelines for submitting code & software for further information.

Data

Policy information about availability of data

All manuscripts must include a data availability statement. This statement should provide the following information, where applicable:

- Accession codes, unique identifiers, or web links for publicly available datasets
- A description of any restrictions on data availability
- For clinical datasets or third party data, please ensure that the statement adheres to our policy

RNA-seq data have been deposited at GEO and is publicly available as of the date of publication under the following accession number: GSE186785.
Accession tokens will be generated upon request from the editorial board and/or reviewers.

Field-specific reporting

Please select the one below that is the best fit for your research. If you are not sure, read the appropriate sections before making your selection.

- [x] Life sciences
- [ ] Behavioural & social sciences
- [ ] Ecological, evolutionary & environmental sciences

For a reference copy of the document with all sections, see nature.com/documents/nr-reporting-summary-flat.pdf

Life sciences study design

All studies must disclose on these points even when the disclosure is negative.

Sample size	No statistical methods were used to predetermine sample size. The sample size for experiments was selected based on variance observed in prior experiments of a similar nature, as well as practical considerations. Practical considerations include cost of experiments, space constraints, and time required to process the experiments.
Data exclusions	DRCs in worms knocked-down for genes involved in the biosynthesis of fatty acids were realized with 2 more doses, 62.5 and 125 µM, respectively (Supplementary Fig. 2), than the ones realized in animals knocked-down for genes involved in the degradation of fatty acids (Supplementary Fig. 3). We chose to exclude these two doses for the calculation of the AUCs presented in Fig. 2e, in order to permit an unbiased comparison with the AUCs presented in Fig. 2f. Notably, the exclusion of these two doses doesn't induce any change of the structure of the dataset in Fig. 2e.<br><br>No other data presented in the manuscript were excluded from analysis.
Replication	All the results presented in the manuscript are representative of one out of -at least- three biological replicates for imaging results, or result from the quantification of -at least- three independent experiments. The reproducibility of the results obtained in the screening of 1,495 RNAi conditions (Fig. 2) was ensured by retesting the hits quantitatively by RNAi, and testing the drug toxicity in mutant animals when possible (i.e. in Δdhs-19 animals).<br><br>All attempts at replication were successful. And the results obtained were coherent with those observed in the litterature when available (i.e. Effect of tamoxifen in ER+ and ER- cells; Fatty Acid composition of E. coli and B. subtilis, as well as C. elegans animals fed E. coli).
Randomization	No formal randomization method was required or used for this study.
Blinding	Cell culture experiments did not require blinding because SYTOX signals were directly linked with the data during the analysis.<br>Bacteria growth experiments did not require blinding because OD600 measurements were directly linked with the data during the analysis.<br><br>For the experiments aimed at cross-comparing the effect of bacterial diet on the tamoxifen toxicity, no blinding was possible as E. coli, C. aquatica and B. subtilis generate bacterial lawns that are easy to distinguish (e.g. Fig. 1 and Fig. 3).<br><br>For the screening of the E. coli mutant library (Fig 2) and of the 1,495 RNAi conditions (Fig 3), as well as for the retest by RNAi of the fatty acid biosynthesis and fatty acid degradation pathways (Fig. 2), RNAi conditions were named after their location in the RNAi library (e.g. Plate_1-A01, Plate_2-H12). The identity of the genes was only revealed after data were collected and analyzed.<br><br>For the screening of the C. aquatica library: the library is not ordered and the identity of the hits can only be revealed by genotyping after data have been collected and analyzed (Fig 2).<br><br>Conditions involving NGM supplements were named following a letter code (e.g. Fig. 5), so that the data were collected and analyzed with no a priori regarding the nature of the conditions. The letter code was used to reveal the identity of the samples after they were collected and analyzed.<br><br>For experiments involving the comparison of C. elegans different strains, the experiments were performed by one individual only which together with the small size of the experiments, couldn't allow for a reliable bldning. The same applies for RNAseq hits retest (e.g. Fig 6).<br><br>For the GC-MS experiments: no blinding was possible during sample collection because bacteria lawns are easy to distinguished.<br>For lipid profiles: Bliding was unnecessary as lipid profiles are extremely different from a species to another, and easy to affiliate to sample groups by eye.<br>For tamoxifen accumulation: letter/number codes were used to reveal the identity of the conditions after the data were analyzed.

Reporting for specific materials, systems and methods

We require information from authors about some types of materials, experimental systems and methods used in many studies. Here, indicate whether each material, system or method listed is relevant to your study. If you are not sure if a list item applies to your research, read the appropriate section before selecting a response.

Materials & experimental systems

n/a	Involved in the study
☒	☐ Antibodies
☐	☒ Eukaryotic cell lines
☒	☐ Palaeontology and archaeology
☐	☒ Animals and other organisms
☒	☐ Human research participants
☒	☐ Clinical data
☒	☐ Dual use research of concern

Methods

n/a	Involved in the study
☒	☐ ChIP-seq
☒	☐ Flow cytometry
☒	☐ MRI-based neuroimaging

Eukaryotic cell lines

Policy information about cell lines

Cell line source(s)	T-47D cells were obtained from Leslie Shaw's lab (UMass Chan Medical School), who was acknowledged in the manuscript. MDA-MB-231 cells were obtained from Michael Lee's lab (UMass Chan Medical School), who is co-author in this manuscript. Cell lines were originally obtained from ATCC.
Authentication	Cell lines used in this study are authentified using STR profiling (ATCC).
Mycoplasma contamination	Cell lines used in this study were tested negative for mycoplasma contaminations before freezing, and regularly once in culture, using MycoAlert Mycoplasma Detection Kit manufactured by Lonza. Growing cultures were kept at low passage number.
Commonly misidentified lines (See ICLAC register)	No commonly misidentified cell lines were used in the study.

Animals and other organisms

Policy information about studies involving animals; ARRIVE guidelines recommended for reporting animal research

Laboratory animals	C. elegans was kept at low passage number. Strains used in this study: N2 Bristol, ced-3 mutant (n717), ced-4 mutants (n1162), dhs-19 mutants (VL1313)
Wild animals	No wild animals were used in the study.
Field-collected samples	No field-collected samples were used in the study.
Ethics oversight	The study did not require an ethical approval.

Note that full information on the approval of the study protocol must also be provided in the manuscript.
